# Comparative Genomics Reveals the Evolutionary Expansion and Diversification of the NPF Gene Family in Grasses

**DOI:** 10.3390/genes17060688

**Published:** 2026-06-11

**Authors:** Qian Zhang, Xiangling Zeng, Keting Zhao, Jingjing Zou, Xuan Cai, Yingting Zhang, Zeqing Li, Xusheng Gong, Yuanhang Wu, Shian Cao, Hongguo Chen, Jie Yang, Wenjie Xia

**Affiliations:** 1 Key Laboratory of National Forestry and Grassland Administration on Osmanthus Fragrans, School of Nuclear Technology and Chemistry & Biology, Hubei University of Science and Technology, Xianning 437100, China; qianzhang@hbust.edu.cn (Q.Z.);; 2 Key Laboratory of Molecular Microbiology and Technology, Ministry of Education, College of Life Sciences, Nankai University, Tianjin 300071, China

**Keywords:** *NPF* gene family, grasses evolution, whole-genome duplication, comparative genomics, RNA-seq

## Abstract

**Background/Objectives**: NPF proteins are important transporters that mediate nitrate uptake, nutrient allocation, and abiotic stress responses in plants. However, the evolutionary patterns of the *NPF* gene family in grasses remain largely unclear. This study aimed to clarify the evolutionary expansion and stress response characteristics of *NPF* genes in Poaceae. **Methods**: A comprehensive comparative genomic analysis was conducted across nine representative Poaceae species and *Arabidopsis thaliana*. Multiple analytical approaches were used, including gene family identification, phylogenetic classification, collinearity analysis, Ka/Ks calculation, cis-element prediction, protein interaction analysis, and RNA-seq expression verification. **Results**: A total of 1109 *NPF* genes were identified with substantial variation in gene copy number among species, particularly the remarkable expansion observed in hexaploid *Triticum aestivum*. Phylogenetic analysis classified grass NPF proteins into eight major subfamilies, while collinearity analyses revealed that whole-genome duplication and segmental duplication were the primary drivers of NPF expansion. Most duplicated gene pairs exhibited Ka/Ks values below 1, indicating strong purifying selection during evolution. Promoter analyses identified abundant stress- and hormone-responsive cis-elements, especially in Triticeae species. In addition, protein–protein interaction and RNA-seq analyses suggested potential functional associations among *NPF* genes and revealed expression variation under low-temperature treatments in rice and wheat. **Conclusions**: Collectively, this study objectively clarifies the evolutionary expansion, functional conservation, and potential stress-responsive diversification of the *NPF* gene family in grasses. These findings provide straightforward and reliable insights for further evolutionary and functional research on the *NPF* gene family in Poaceae.

## 1. Introduction

Nitrogen is one of the most essential macronutrients required for plant growth and development, and nitrate (NO^3−^) represents the predominant nitrogen source absorbed by most terrestrial plants. Efficient nitrate uptake, transport, storage, and redistribution are therefore critical for plant productivity, nitrogen use efficiency (NUE), and environmental adaptation [1,2,3,4]. In plants, nitrate transport is mainly mediated by three transporter families, including *NRT1/NPF*, *NRT2*, and *NRT3*, among which the NRT1/Peptide Transporter Family (*NPF*) constitutes the largest and most functionally diverse transporter group [5,6,7,8,9,10,11,12,13]. Initially characterized as low-affinity nitrate transporters, NPF proteins were subsequently found to transport a broad spectrum of substrates, including peptides, phytohormones, glucosinolates, chloride ions, and secondary metabolites, indicating remarkable functional diversification during plant evolution [14,15,16].

Members of the *NPF* are widely distributed in higher plants and generally encode membrane-localized proteins containing 10–14 transmembrane domains, with most members possessing 12 conserved transmembrane helices characteristic of the major facilitator superfamily (MFS) transporters [17]. Based on phylogenetic relationships, plant NPF proteins are commonly classified into eight major subfamilies (*NPF1*–*NPF8*), each exhibiting distinct substrate preferences and biological functions [14]. In *Arabidopsis thaliana*, several *NPF* members have been extensively characterized. For example, *AtNPF6.3/AtNRT1.1* functions as both a dual-affinity nitrate transporter and nitrate sensor involved in nitrate signaling and root development [18,19,20], whereas *AtNPF7.3/AtNRT1.5* mediates root-to-shoot nitrate transport through xylem loading [21,22]. In addition, *AtNPF7.2/AtNRT1.8* regulates nitrate unloading from the xylem under stress conditions and contributes to nitrate redistribution within plants [23]. In rice, several *NPF* genes, including *OsNPF6.5* and *OsNPF7.3*, have been implicated in nitrate uptake, long-distance transport, nitrogen utilization efficiency, and abiotic stress-associated responses, highlighting the multifunctional roles of NPF transporters in integrating nutrient and environmental signaling pathways [24,25,26,27,28].

With the rapid development of plant genomics, genome-wide analyses of the *NPF* have been performed in multiple plant species, including *A. thaliana*, rice, maize, tea plant, cassava, sugarcane, and sorghum [29,30,31,32,33,34,35,36,37]. These studies demonstrated that the *NPF* underwent extensive expansion and functional diversification during plant evolution, frequently associated with whole-genome duplication (WGD), tandem duplication, and lineage-specific gene retention. In grasses (Poaceae), recurrent polyploidization and chromosomal rearrangement events have profoundly shaped genome evolution and contributed to the expansion of many transporter- and stress-associated gene families [38,39]. For example, the allohexaploid wheat genome experienced multiple rounds of genome hybridization and duplication, resulting in extensive retention of duplicated loci [40,41]. However, despite the agricultural importance of grasses, a comprehensive evolutionary analysis of the *NPF* across representative Poaceae species is still lacking. In particular, the evolutionary conservation, lineage-specific diversification, duplication patterns, regulatory evolution, and stress-responsive expression dynamics of *NPF* genes across grasses remain poorly understood.

In the present study, we performed a comprehensive comparative genomic analysis of the *NPF* gene family across nine representative grass species and *A. thaliana*. A systematic investigation was conducted, including gene identification, phylogenetic classification, physicochemical characterization, gene duplication and synteny analyses, cis-regulatory element prediction, protein–protein interaction network construction, and transcriptomic profiling under abiotic stress conditions. Our results revealed extensive expansion and diversification of the *NPF* in grasses, particularly in Triticeae species, primarily driven by whole-genome duplication and segmental duplication events. In addition, comparative promoter and RNA-seq analyses suggested potential associations between *NPF* genes and stress-responsive regulatory pathways, especially under low-temperature conditions. Collectively, this study provides a comprehensive evolutionary framework for understanding the origin, expansion, and functional diversification of the *NPF* in grasses and offers valuable candidate genes for future functional studies and molecular breeding aimed at improving nutrient utilization and stress tolerance in cereal crops.

## 2. Materials and Methods

### 2.1. Identification of NPF Members in Representative Grass Genomes

To investigate the evolutionary characteristics of the *NPF* gene family in grasses, genome sequences and corresponding annotation files of representative Poaceae species, including *Brachypodium distachyon*, *Oryza sativa*, *Triticum aestivum*, *Triticum urartu*, *Aegilops tauschii*, *Hordeum vulgare*, *Zea mays*, *Sorghum bicolor* and *Setaria italica* were collected from publicly available genome databases, including Phytozome and Ensembl Plants [42]. The genome and annotation datasets of *Arabidopsis thaliana* were retrieved from TAIR and incorporated as a dicot reference species for comparative analyses [43].

Candidate NPF proteins were identified using a combined HMM- and homology-based strategy. The conserved NPF domain profile (PF00854) was downloaded from the Pfam database and employed as a query for HMMER v3.3.2 searches with an E-value cutoff of 1 × 10^−10^ [44]. To further improve identification accuracy, known *A. thaliana* NPF protein sequences were additionally used for BLASTP searches against all analyzed proteomes with an E-value cutoff of 1 × 10^−10^ [45]. Only candidate proteins identified by both HMMER (v3.0) and BLASTP approaches were retained for further analyses. Candidate proteins were subsequently screened for the presence of complete conserved NPF domains using both hmmscan and Pfam-scan, and proteins lacking the conserved PF00854 domain were excluded. For genes producing multiple splice variants, only the longest protein isoform was retained for subsequent analyses.

### 2.2. Evolutionary Classification and Phylogenetic Reconstruction of NPF Proteins

Full-length amino acid sequences of all identified NPF proteins were aligned using MUSCLE v3.8 [46]. The aligned sequences were subsequently used to reconstruct maximum-likelihood phylogenetic trees with IQ-TREE v2.0.3 [47]. Branch robustness was assessed through 1000 bootstrap replicates. Based on phylogenetic topology and evolutionary relationships, NPF proteins were further classified into distinct subfamilies. The final phylogenetic trees were visualized and annotated using the R package ggtree (v3.10.0) [48,49].

### 2.3. Analysis of Chromosomal Distribution, Gene Duplication, and Synteny Relationships

The physical locations of NPF genes were obtained from genome annotation files and mapped onto chromosomes to investigate genomic distribution patterns. The density and clustering characteristics of NPF genes were further analyzed across different grass genomes. Gene duplication events, including tandem duplication and segmental/WGD, were identified using MCScanX (v1.1) [4]. To explore evolutionary conservation of NPF loci among grasses, comparative synteny analyses were carried out using JCVI [50]. Collinear relationships within and between species were visualized using JCVI and the circlize package in R [51]. Evolutionary selection pressure acting on duplicated gene pairs was estimated by calculating synonymous substitution rates (Ks), nonsynonymous substitution rates (Ka), and Ka/Ks ratios using KaKs_Calculator 2.0 [52].

### 2.4. Prediction of Cis-Acting Regulatory Elements in NPF Promoters

To evaluate the potential regulatory characteristics of NPF genes, 2 kb genomic regions upstream of transcription start sites were extracted and defined as putative promoter sequences. Cis-acting regulatory elements were identified using the PlantCARE database [53]. Comparative enrichment analyses of cis-elements among species were subsequently performed, and the distribution of regulatory elements was visualized using R-based plotting tools.

### 2.5. Construction of Protein Interaction Networks for NPF Proteins

To predict functional associations among NPF proteins, protein–protein interaction networks were generated based on the AraNet2 platform [54]. Only interactions with a confidence weight score ≥ 4 were retained for downstream analyses. Functional annotation and orthology assignment of interacting proteins were conducted using EggNOG-mapper (v2.1.12) [55]. The interaction networks were further processed and visualized using the R package ggNetView (v1.0) (https://github.com/Jiawang1209/ggNetView (accessed on 10 March 2026)) to explore network topology and identify highly connected nodes within the predicted NPF interaction networks.

### 2.6. Transcriptome Analysis of NPF Genes Under Abiotic Stress Conditions

RNA-seq data used for tissue-specific expression analysis were retrieved from the NCBI SRA database under accession number PRJNA772921 and PRJNA787922. Raw FASTQ files were aligned to reference genomes using HISAT2 (v2.2.0) [56]. Gene-level expression counts were quantified using featureCounts (v1.6.4) [57]. Differential expression analysis was conducted using the DESeq2 package (v1.48.2) in R. Raw count matrices generated by featureCounts were used as input. Genes with an adjusted *p*-value (FDR) < 0.05 and |log2FoldChange| > 1 were considered significantly differentially expressed. The resulting read counts were converted to FPKM values. For visualization purposes, FPKM values were standardized using row-wise Z-score normalization implemented in the R package pheatmap (scale = “row”). The resulting heatmaps were used to compare relative expression patterns of NPF genes under different treatment conditions.

## 3. Results

### 3.1. Genome-Wide Identification and Physicochemical Characterization of NPF Gene Family in Grasses

**Figure 1:**
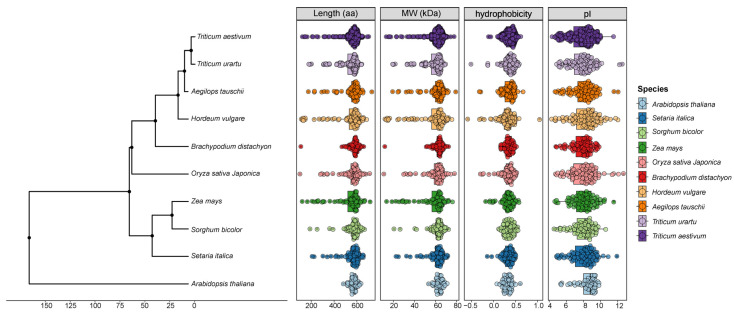
Identification and physicochemical characterization of NPF members in 10 species investigated in this study, including protein length, molecular weight, hydrophilicity and isoelectric point.

Genome-wide identification of the *NPF* gene family revealed a total of 1109 *NPF* genes across nine representative grass species and *Arabidopsis thaliana* (Figure 1, Supplemental Data S1), including *Triticum aestivum* (331), *Triticum urartu* (113), *Aegilops tauschii* (98), *Setaria italica* (93), *Hordeum vulgare* (88), *Sorghum bicolor* (88), *Oryza sativa Japonica* (87), *Zea mays* (80), *Brachypodium distachyon* (78), and *A. thaliana* (53). Considerable variation in *NPF* gene copy number was observed among species. Most diploid grass genomes contained approximately 78–113 *NPF* members, whereas the hexaploid wheat genome possessed 331 *NPF* genes, representing a dramatic expansion of the family. This substantial increase in wheat likely reflects the contribution of polyploidization and large-scale genome duplication events during Triticeae evolution. In contrast, the relatively conserved *NPF* size among diploid grasses suggests that the overall *NPF* repertoire remained evolutionarily stable following divergence of major Poaceae lineages.

Despite differences in gene copy number, the physicochemical characteristics of NPF proteins were generally conserved across species. Protein lengths ranged from 108 to 732 amino acids, with average lengths predominantly distributed between approximately 525 and 576 amino acids. Similarly, predicted molecular weights ranged from ~11.8 to 79.2 kDa, whereas average molecular weights were consistently centered around 56–63 kDa in all analyzed species. These results are consistent with the highly conserved structural characteristics previously reported for plant NPF transporters, most of which contain multiple transmembrane domains and belong to the major facilitator superfamily.

Hydrophobicity analysis indicated that most NPF proteins were moderately hydrophobic, with average GRAVY values ranging from 0.22 to 0.34, reflecting the membrane-associated nature of NPF transporters. In addition, theoretical isoelectric points (pI) varied substantially among species, ranging from approximately 4.28 to 12.06. Compared with *A. thaliana*, grass species exhibited broader pI distributions, especially in Triticeae genomes, implying an increase in the biochemical diversity of NPF proteins during grass evolution. Collectively, these findings suggest that expansion of the *NPF* in grasses occurred while maintaining highly conserved core structural and physicochemical features, supporting the functional conservation of *NPF* transporters during Poaceae diversification.

### 3.2. Phylogenetic Classification and Evolutionary Distribution of NPF Gene Family in Grasses

**Figure 2:**
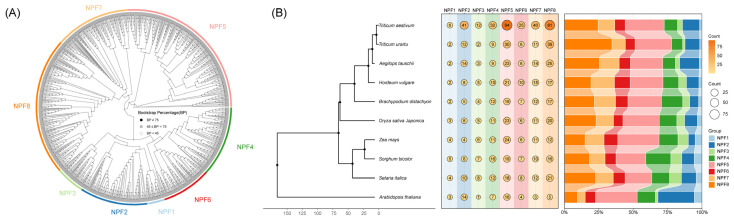
Phylogenetic classification and evolutionary diversification of NPF proteins in grasses. (**A**) Maximum-likelihood phylogenetic tree constructed using NPF protein sequences from nine representative Poaceae species and Arabidopsis thaliana. (**B**) Comparative distribution of NPF subgroups among different species.

To investigate the evolutionary relationships of NPF proteins in grasses, a maximum-likelihood phylogenetic tree was constructed using NPF protein sequences from nine representative Poaceae species together with *A. thaliana* as an outgroup (Figure 2A). Based on phylogenetic topology and branch support, all NPF proteins were classified into eight major subfamilies, designated *NPF1*–*NPF8*, which is consistent with the established classification system of plant NPF transporters. All eight subfamilies were detected in both grasses and *A. thaliana*, indicating that the major *NPF* lineages were already established prior to the divergence of monocots and dicots. Among these subfamilies, *NPF5* represented the largest clade, whereas *NPF1* and *NPF2* contained comparatively fewer members, suggesting unequal evolutionary expansion among different *NPF* lineages.

Although all analyzed grass species retained representatives from each *NPF* subgroup, substantial variation in subfamily composition was observed among species (Figure 2B). *NPF5*, *NPF6*, and *NPF8* constituted the dominant subfamilies in most grass genomes, implying that these groups experienced extensive retention and amplification during grass evolution. In contrast, *NPF1* and *NPF2* remained relatively small and evolutionarily conserved across species. Notably, the hexaploid wheat genome (*T. aestivum*) exhibited remarkable expansion in nearly all subfamilies, particularly within *NPF5* and *NPF8*, whereas diploid grasses displayed more balanced subgroup distributions. Similar but less pronounced expansion patterns were also observed in Triticeae species such as *T. urartu* and *A. tauschii*.

A comparative analysis of subfamily composition further revealed that closely related grass species tended to share similar *NPF* distribution patterns, reflecting strong phylogenetic conservation during Poaceae diversification. However, the relative abundance of individual subfamilies varied substantially among lineages, suggesting that lineage-specific duplication and differential gene retention contributed to evolutionary divergence among *NPF* gene lineages in grasses. Collectively, these results indicate that the evolutionary expansion of the *NPF* in Poaceae was highly asymmetric among subfamilies and was strongly influenced by polyploidization and genome evolution.

### 3.3. Gene Duplication Patterns and Intraspecies Collinearity Analysis of the NPF Gene Family

**Figure 3:**
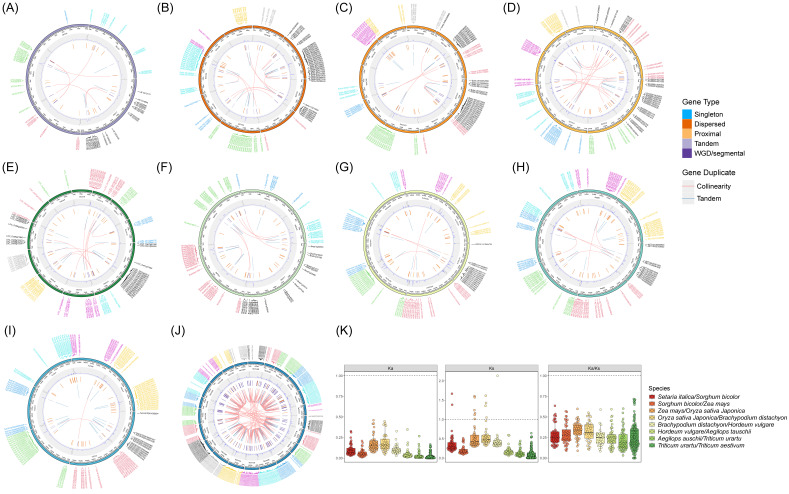
Intra-species collinearity and duplication patterns of NPF genes in grasses. Circos plots showing chromosomal distribution, duplication types, and intra-species collinearity relationships of NPF genes in representative plant species. (**A**) Arabidopsis thaliana; (**B**) Setaria italica; (**C**) Sorghum bicolor; (**D**) Zea mays; (**E**) Oryza sativa Japonica; (**F**) Brachypodium distachyon; (**G**) Hordeum vulgare; (**H**) Aegilops tauschii; (**I**) Triticum urartu and (**J**) Triticum aestivum. (**K**) Distribution of Ka, Ks, and Ka/Ks values for tandem duplicated and WGD-derived NPF gene pairs across different species.

To investigate the evolutionary expansion mechanisms of the *NPF* gene family in grasses, intraspecies collinearity and gene duplication analyses were performed across nine representative Poaceae species and *A. thaliana* (Figure 3, Supplemental Data S2). Extensive collinear relationships were detected in all analyzed genomes, indicating that large-scale duplication events contributed substantially to the expansion of the *NPF* during grass evolution. Compared with diploid species, Triticeae genomes, particularly hexaploid *T. aestivum*, exhibited markedly more complex collinearity networks and contained a substantially larger number of duplicated *NPF* genes. This pattern is consistent with the extensive genome duplication and polyploidization history of wheat evolution.

*NPF* genes were classified into several duplication categories, including singleton, dispersed, proximal, tandem, and whole-genome duplication/segmental duplication (WGD/segmental). Among these categories, WGD/segmental duplication represented the predominant duplication mode in most grass species, especially in Triticeae genomes, where numerous *NPF* genes were retained within large collinear chromosomal blocks. In contrast, tandem duplication events were comparatively fewer and mainly involved physically adjacent genes with highly similar gene identifiers located on the same chromosome. By comparison, WGD-derived *NPF* genes were generally distributed across different chromosomes or distant genomic regions, indicating that large-scale chromosomal duplication events rather than local tandem amplification primarily drove expansion of the *NPF* in grasses.

Considerable variation in duplication patterns was also observed among species. Diploid grasses such as *B. distachyon*, *S. italica*, and *S. bicolor* displayed relatively moderate numbers of duplicated *NPF* genes and simpler collinearity structures, whereas wheat exhibited extensive retention of duplicated loci across nearly all chromosomes. Similar but less pronounced patterns were also identified in *T. urartu* and *A. tauschii*, supporting the idea that progressive genome expansion within Triticeae lineages promoted continuous accumulation of NPF members. Furthermore, Ka/Ks analysis showed that most duplicated gene pairs exhibited Ka/Ks ratios below 1 (Figure 3K), suggesting that the majority of *NPF* duplicated genes have undergone strong purifying selection during evolution. Collectively, these findings indicate that whole-genome duplication and segmental duplication were the major driving forces underlying expansion and evolutionary history of the *NPF* in grasses.

### 3.4. Interspecies Collinearity and Evolutionary Conservation of the NPF Gene Family

**Figure 4:**
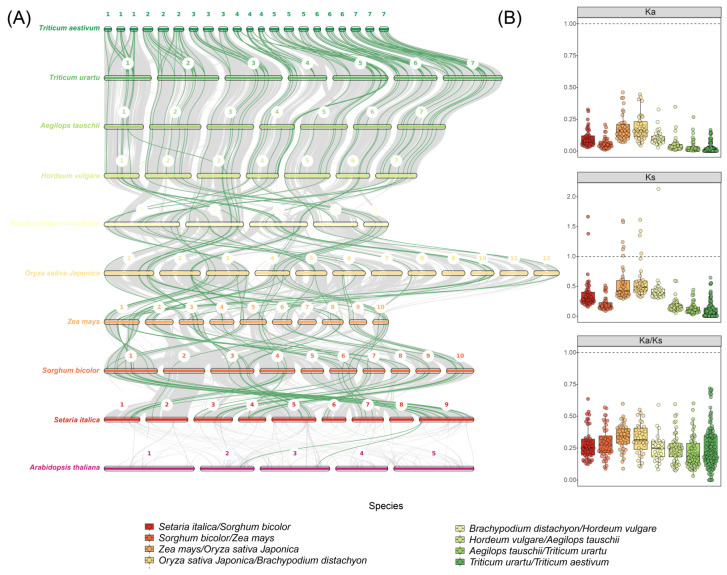
Comparative synteny and evolutionary conservation of NPF genes among representative grass species. (**A**) Cross-species collinearity relationships of NPF genes among representative Poaceae species and Arabidopsis thaliana. (**B**) Distribution of Ka, Ks, and Ka/Ks values of orthologous NPF gene pairs among different species combinations.

To further investigate the evolutionary conservation of the *NPF* gene family among grasses, comparative interspecies synteny analyses were performed across representative Poaceae species and *A. thaliana* (Figure 4A, Supplemental Data S3). Extensive collinear relationships were identified among closely related grass genomes, indicating that a large proportion of *NPF* genes were evolutionarily conserved during Poaceae diversification. In total, 746 orthologous *NPF* gene pairs were identified among the analyzed species, although the number of conserved pairs varied substantially across different evolutionary lineages. The largest number of syntenic gene pairs was detected between *T. aestivum* and *T. urartu* (246 pairs), followed by *A. tauschii*/*H. vulgare* (86 pairs), *Z. mays*/*S. bicolor* (81 pairs), *S. bicolor*/. (76 pairs), and *A. tauschii*/*T. urartu* (75 pairs). In contrast, only six collinear *NPF* gene pairs were detected between *S. italica* and *A. thaliana*, reflecting the large evolutionary divergence between monocot and dicot lineages.

The distribution of orthologous gene pairs strongly corresponded to phylogenetic relationships among species. Closely related Triticeae species displayed the highest degree of syntenic conservation, whereas more distantly related grass species exhibited progressively reduced numbers of conserved *NPF* loci. These results suggest that lineage divergence, chromosomal rearrangements, and differential gene retention collectively shaped the current distribution of *NPF* genes during grass evolution. Notably, Triticeae genomes exhibited highly interconnected collinearity networks, supporting the hypothesis that recurrent polyploidization and large-scale genome duplication events contributed substantially to expansion and retention of *NPF* genes in wheat-related species.

To evaluate evolutionary selection pressure acting on orthologous *NPF* gene pairs, Ka, Ks, and Ka/Ks ratios were calculated for all interspecies collinear pairs (Figure 4B). Most orthologous gene pairs exhibited Ka/Ks values below 1, indicating that *NPF* genes have predominantly undergone strong purifying selection during grass evolution. Although a small number of gene pairs displayed elevated Ka/Ks values, the overall evolutionary pattern suggests that functional conservation has been maintained across most *NPF* orthologs following species divergence. Collectively, these findings demonstrate that the *NPF* gene family exhibits strong evolutionary conservation among grasses while simultaneously undergoing lineage-specific expansion and diversification during Poaceae evolution.

### 3.5. Cis-Regulatory Element Analysis of NPF Gene Family

**Figure 5:**
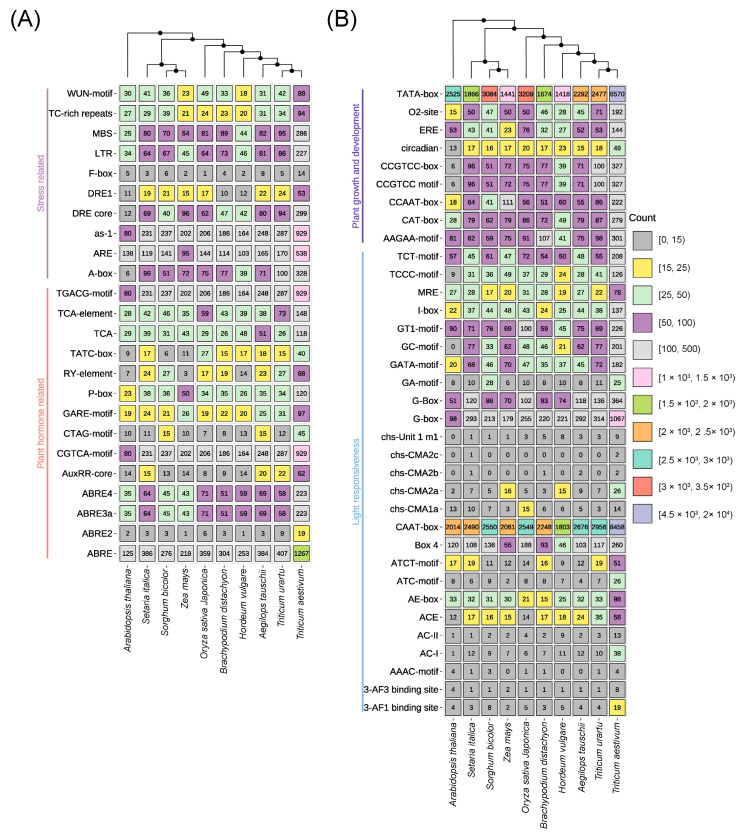
Comparative analysis of cis-regulatory elements in NPF gene promoters across representative plant species. (**A**) Elements related to stress and plant hormones. (**B**) Elements involved in plant growth, development and light response.

To explore the potential regulatory mechanisms of *NPF* genes in grasses, cis-acting regulatory elements within the 2 kb upstream promoter regions were systematically analyzed across nine representative Poaceae species and *A. thaliana* (Figure 5). A large number of stress-responsive, hormone-responsive, light-responsive, and growth- and development-related cis-elements were identified in *NPF* promoters, indicating that *NPF* genes may participate in diverse biological and environmental response pathways. Overall, Triticeae species, particularly *T. aestivum*, possessed substantially more cis-elements than diploid grass species, consistent with the large expansion of the *NPF* gene family in wheat following polyploidization.

Among stress- and hormone-related elements (Figure 5A), ABRE, associated with abscisic acid (ABA) responsiveness, represented the most abundant cis-element in nearly all species. The highest number of ABRE elements was detected in *T. aestivum* (approximately 1850), whereas comparatively fewer ABREs were observed in *A. thaliana* and diploid grasses. In addition, several stress-responsive elements, including ARE, MBS, LTR, and DRE-related motifs, were widely distributed among grass species, suggesting that *NPF* genes may possess the regulatory potential to response to abiotic stress, particularly responses to drought, low temperature, and anaerobic stress. Notably, low-temperature-responsive LTR elements and dehydration-responsive DRE motifs were especially enriched in Triticeae genomes, suggesting that expansion of *NPF* genes in wheat-related species may have increased the diversity of predicted regulatory elements associated with environmental responses.

Light-responsive and growth-related cis-elements were also highly enriched in *NPF* promoters (Figure 5B). Elements such as G-box, Box 4, GT1-motif, and TATA-box were broadly distributed across all analyzed species, indicating that *NPF* gene expression may be strongly influenced by light signaling and transcriptional regulation. Among these motifs, TATA-box and CAAT-box represented the two most abundant core promoter elements in all species, reflecting highly conserved transcriptional regulatory structures of *NPF* genes during grass evolution. Furthermore, Triticeae species exhibited markedly higher numbers of light-responsive and development-associated cis-elements than diploid grasses, again supporting the idea that polyploidization promoted expansion not only of *NPF* gene copy number but also of promoter regulatory complexity. Collectively, these results suggest that *NPF* genes in grasses possess highly diversified cis-regulatory architectures and may be associated with nutrient transport, growth regulation, and abiotic stress-related regulatory pathways.

### 3.6. Protein–Protein Interaction Network Analysis of NPF Proteins

**Figure 6:**
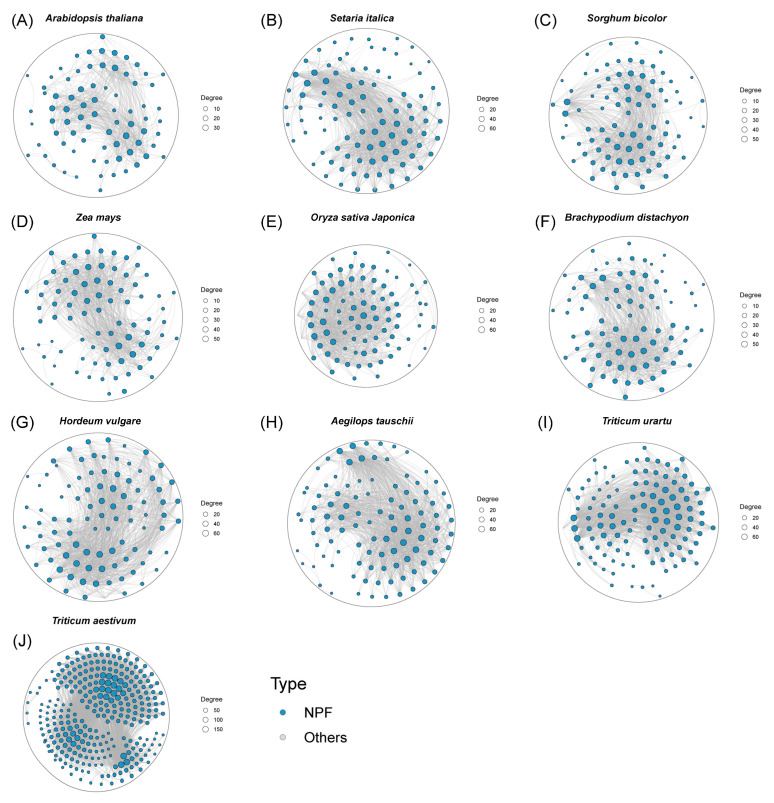
Functional interaction networks of NPF proteins across representative plant species. (**A**) Arabidopsis thaliana; (**B**) Setaria italica; (**C**) Sorghum bicolor; (**D**) Zea mays; (**E**) Oryza sativa Japonica; (**F**) Brachypodium distachyon; (**G**) Hordeum vulgare; (**H**) Aegilops tauschii; (**I**) Triticum urartu; and (**J**) Triticum aestivum.

To further explore the potential functional relationships among NPF proteins, protein–protein interaction (PPI) networks were constructed for nine representative grass species and *A. thaliana* (Figure 6). The predicted interaction networks suggested potential functional associations among NPF proteins in all analyzed species, suggesting that members of the *NPF* may be functionally associated with common transport and signaling pathways. Notably, most NPF proteins were directly or indirectly connected with other *NPF* members, indicating potential connectivity among NPF members.

The complexity of the interaction networks varied substantially among species and generally corresponded to differences in *NPF* gene family size. Diploid grass species such as *B. distachyon*, *S. italica*, and *S. bicolor* displayed relatively moderate and compact interaction networks, whereas Triticeae species, especially hexaploid *T. aestivum*, exhibited highly dense and complex interaction patterns. In wheat, numerous NPF proteins showed high connectivity (high-degree nodes), suggesting that expansion of the *NPF* following polyploidization may have increased the complexity of the predicted interaction networks among NPF proteins.

In addition to extensive NPF–NPF interactions, several NPF proteins also interacted with non-NPF proteins, implying potential functional associations between NPF transporters and other biological pathways. However, the majority of central hub proteins within the networks were still composed of *NPF* members, further supporting the idea that NPF proteins may participate in related transport and signaling pathways during plant growth and environmental adaptation. Collectively, these findings suggest that the *NPF* possesses numerous predicted functional associations in grasses, and that expansion of *NPF* genes during Poaceae evolution may have contributed not only to increased gene copy number but also to greater complexity of predicted interaction networks.

### 3.7. Expression Profiles of NPF Genes Under Low-Temperature Stress

**Figure 7:**
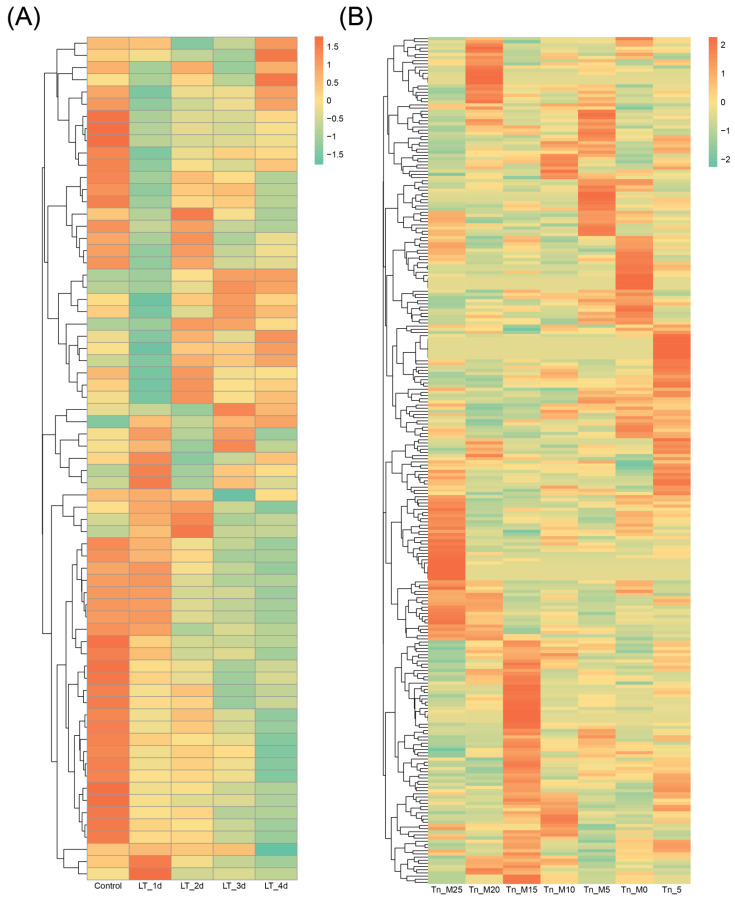
Transcriptional responses of NPF genes under low-temperature stress in rice and wheat. (**A**) LT_1d, LT_2d, LT_3d, and LT_4d). (**B**) Heatmap showing expression patterns of NPF genes in Triticum aestivum under different low-temperature conditions (Tn_M25 to Tn_5).

To investigate the potential roles of *NPF* genes in low-temperature adaptation, RNA-seq datasets from rice and wheat subjected to cold stress were analyzed (Figure 7). Overall, a substantial proportion of *NPF* genes displayed expression variation under low-temperature treatments, suggesting potential associations between *NPF* gene expression patterns and cold-related conditions in grasses. Both rice and wheat displayed dynamic expression changes following low-temperature exposure, although the overall transcriptional patterns differed between the two species.

In *O. sativa*, multiple *NPF* genes displayed relatively higher transcript abundance during the early stages of low-temperature treatment (LT_1d and LT_2d), whereas expression of several genes gradually decreased during prolonged stress exposure (LT_3d and LT_4d) (Figure 7A). These expression patterns indicate that different rice *NPF* genes exhibited distinct transcriptional profiles under low-temperature treatments. In addition, hierarchical clustering analysis revealed that different *NPF* members displayed highly diverse expression profiles, indicating transcriptional divergence among duplicated *NPF* genes under the analyzed conditions.

Compared with *O. sativa*, *T. aestivum* exhibited a broader and more complex transcriptional response under low-temperature conditions (Figure 7B). A large number of *TaNPF* genes exhibited elevated transcript abundance under extremely low-temperature conditions, particularly under −25 °C and −20 °C treatments, whereas other genes maintained relatively stable or reduced expression levels. Notably, several *NPF* genes displayed sustained high expression across multiple low-temperature conditions, suggesting that these genes may represent promising candidates for future studies of cold-associated responses in wheat. The larger number of *NPF* genes exhibiting expression variation under low-temperature treatments in wheat is likely associated with extensive expansion of the *NPF* following polyploidization and genome duplication. Using Control as the reference condition, differential expression analysis identified 55 significant differential expression records corresponding to 23 unique *NPF* genes in rice under low-temperature treatments (LT_1d, LT_2d, LT_3d, and LT_4d). In wheat, using Tn_5 as the reference condition, 251 significant differential expression records corresponding to 89 unique *TaNPF* genes were detected across the examined temperature treatments. These results indicate that a subset of *NPF* genes exhibited statistically supported transcriptional responses to low-temperature conditions. The complete differential expression results are provided in Supplemental Data S4. Collectively, these findings indicate that numerous *NPF* genes exhibit distinct expression patterns under low-temperature treatments and may be associated with transcriptional responses to cold-related conditions in grasses.

## 4. Discussion

The NPF gene family is one of the most important transporter families involved in nitrate uptake, translocation, and nutrient allocation in plants. Previous studies demonstrated that plant NPF proteins originated early during land plant evolution and subsequently underwent extensive diversification associated with nutrient adaptation and environmental responses [14,15]. In the present study, comparative genomic analyses across nine representative grass species and Arabidopsis thaliana revealed substantial variation in NPF gene copy number among lineages, with the hexaploid wheat genome containing dramatically more NPF genes than diploid grasses. Similar expansion patterns have been reported for many transporter-related gene families in polyploid crops, indicating that recurrent whole-genome duplication (WGD) and polyploidization are major driving forces underlying gene family amplification during grass evolution [38,58]. Despite extensive expansion, the physicochemical properties of NPF proteins remained highly conserved across species, particularly regarding protein length, hydrophobicity, and membrane-associated characteristics. These findings are consistent with previous reports showing that most plant NPF transporters contain conserved transmembrane structures essential for nitrate transport activity [5,15]. Therefore, the expansion of the NPF in grasses likely occurred while maintaining core transporter functions required for nitrate uptake and allocation. Beyond simple gene family expansion, the observed retention patterns suggest that NPF genes may have been preferentially preserved during grass genome evolution. NPF transporters play indispensable roles in nitrate uptake, allocation, and signaling, all of which directly influence plant growth and nitrogen use efficiency. According to the gene balance hypothesis, genes involved in interconnected regulatory and transport systems are often preferentially retained following whole-genome duplication events because dosage imbalance may negatively affect network function [1]. The extensive retention of NPF duplicates observed in Triticeae species therefore may reflect both polyploidization history and functional constraints associated with nitrogen transport networks.

Phylogenetic and synteny analyses further indicated that the evolutionary expansion of the grass NPF was highly asymmetric among different subfamilies. Among the eight identified subgroups, NPF5, NPF6, and NPF8 represented the largest and most extensively expanded lineages in grasses, particularly in Triticeae genomes. Previous functional studies demonstrated that members of these subfamilies participate in nitrate transport, long-distance nutrient allocation, and stress adaptation. For example, AtNPF6.3/NRT1.1 functions as a dual-affinity nitrate transporter and nitrate sensor regulating nitrogen signaling pathways [20], whereas rice OsNPF6.5 contributes to nitrate uptake and nitrogen use efficiency [16]. The preferential retention of these subgroups following WGD events therefore suggests that duplicated NPF genes may provide additional genetic resources that could facilitate adaptation under fluctuating environmental and nutritional conditions. This non-random retention pattern suggests that expansion of the NPF was not merely a consequence of genome duplication, but may also reflect selective constraints acting on genes involved in nutrient acquisition and signaling. According to the gene balance hypothesis, genes participating in complex regulatory and transport networks are often preferentially retained after whole-genome duplication because alterations in gene dosage can disrupt network stability [59]. Therefore, the extensive preservation of NPF paralogs in grasses, particularly in Triticeae species, may be associated with the long-term retention of nitrogen transport-related gene networks during grass genome evolution. In contrast, smaller subfamilies such as NPF1 and NPF2 remained relatively conserved across species, implying stronger functional constraints during evolution. Interestingly, the degree of expansion differed substantially among grass lineages. Triticeae species exhibited much stronger expansion across multiple NPF subfamilies than rice, sorghum, or Brachypodium. This pattern is consistent with the complex polyploidization history of Triticeae genomes, particularly bread wheat, which originated through multiple hybridization and genome duplication events [38]. The resulting retention of duplicated NPF genes may have increased the diversity of predicted expression patterns and regulatory architectures among NPF genes. Importantly, both intraspecies and interspecies collinearity analyses demonstrated that WGD/segmental duplication represented the predominant mechanism driving NPF expansion, whereas tandem duplication contributed comparatively less. Similar evolutionary patterns have been observed in cereal genomes, where large-scale chromosomal duplication events played major roles in expansion of stress-responsive and transporter-associated gene families [60,61]. Moreover, most duplicated and orthologous NPF gene pairs exhibited Ka/Ks ratios below 1, indicating that strong purifying selection acted on NPF genes during grass diversification and contributed to long-term functional conservation.

Functional information from experimentally characterized NPF genes further supports the potential significance of the observed expansion patterns. In Arabidopsis, AtNPF6.3 acts as a dual-affinity nitrate transporter and nitrate sensor [20], whereas AtNPF7.3 and AtNPF7.2 mediate root-to-shoot nitrate allocation and nitrate remobilization [21,23]. In rice, OsNPF6.5 contributes to nitrate uptake efficiency and grain productivity [24]. Natural variation in NRT1.1B has been shown to contribute to differences in nitrate-use efficiency among rice subspecies, further highlighting the evolutionary importance of NPF-mediated nitrogen utilization pathways [25]. The presence of large numbers of homologous genes within expanded grass NPF subfamilies suggests that gene duplication may have contributed to increased genetic complexity within the NPF. Nevertheless, additional experimental validation will be required to determine whether these homologs retain ancestral functions or have undergone functional divergence. In addition to genomic expansion, substantial diversification in promoter regulatory architecture and transcriptional responsiveness was observed among grass NPF genes. Triticeae species possessed markedly higher numbers of stress-responsive and hormone-responsive cis-elements, including ABRE, LTR, and DRE-related motifs, than diploid grasses. These results suggest that polyploidization may have increased the diversity of predicted cis-regulatory architectures among NPF promoters, potentially providing additional regulatory potential under different environmental conditions. Consistent with this observation, RNA-seq analyses revealed substantial expression variation among numerous NPF genes under low-temperature treatments in both rice and wheat. Increasing evidence suggests that nitrate transport and nitrogen signaling pathways are closely associated with abiotic stress adaptation, including cold tolerance and stress-induced metabolic regulation [1,62]. In Arabidopsis, nitrate transporters have been shown to participate in hormone signaling and stress-responsive regulatory networks beyond nutrient transport alone [63]. Furthermore, the predicted protein–protein interaction networks identified in this study suggest potential functional associations among NPF proteins and indicate possible coordination within nutrient transport- and signaling-related pathways. Collectively, these findings suggest that expansion of the NPF during Poaceae evolution was accompanied by diversification of predicted regulatory architectures and expression patterns. However, whether these patterns are associated with functional divergence among NPF genes remains to be determined through future experimental studies.

## 5. Conclusions

In this study, we performed a comprehensive comparative genomic and evolutionary analysis of the NPF gene family across nine representative grass species and Arabidopsis thaliana. Our results showed that the NPF underwent extensive expansion during Poaceae evolution, particularly in Triticeae species, and that whole-genome duplication and segmental duplication events were closely associated with this expansion. Despite substantial variation in gene copy number among species, NPF proteins retained highly conserved structural and physicochemical characteristics, indicating strong evolutionary constraints on core transporter functions. Phylogenetic, collinearity, and Ka/Ks analyses further revealed that most NPF genes experienced strong purifying selection during grass diversification. In addition, promoter analyses showed extensive enrichment of stress-and hormone-responsive cis-elements, while protein–protein interaction networks suggested potential functional associations among NPF members. Transcriptome analyses revealed expression variation among numerous NPF genes under low-temperature treatments in both rice and wheat, providing candidate genes for future investigations of expression responses under low-temperature conditions. Overall, these findings provide new insights into the evolutionary dynamics of the NPF gene family in grasses and identify candidate genes for future functional characterization of nutrient utilization and stress-associated processes in cereal crops.

## Data Availability

The original contributions presented in this study are included in the article; further inquiries can be directed to the corresponding authors.

## Supplementary Materials

The following supporting information can be downloaded at: https://www.mdpi.com/article/10.3390/genes17060688/s1, Supplemental Data S1. Analysis of physicochemical properties of NPF gene family in 10 species. Supplemental Data S2. Duplicate gene pairs of NPF gene family in 10 species. Supplemental Data S3. Synteny gene pairs of NPF gene family in 10 species. Supplemental Data S4. The differently expressed genes of RNA-seq in rice and wheat.
